# Carotenoids and Chlorophylls as Antioxidants

**DOI:** 10.3390/antiox9060505

**Published:** 2020-06-09

**Authors:** Antonio Pérez-Gálvez, Isabel Viera, María Roca

**Affiliations:** Food Phytochemistry Department, Instituto de la Grasa (CSIC), University Campus, Building 46, 41013 Sevilla, Spain; aperez@ig.csic.es (A.P.-G.); iviera@ig.csic.es (I.V.)

**Keywords:** ABTS, antioxidant activity, antioxidant capacity, carotenoid, chlorophyll, chlorophyllin, DPPH, LDL, liposomes, Nrf-2

## Abstract

Chlorophylls and carotenoids are natural pigments that are present in our daily diet, especially with the increasing tendency towards more natural and healthy behaviors among consumers. As disturbed antioxidant homeostasis capacities seem to be implicated in the progress of different pathologies, the antioxidant properties of both groups of lipophilic compounds have been studied. The objective of this review was to analyze the state-of-the-art advances in this field. We conducted a systematic bibliographic search (Web of Science™ and Scopus®), followed by a comprehensive and critical description of the results, with special emphasis on highly cited and more recently published research. In addition to an evaluative description of the methodologies, this review discussed different approaches used to obtain a physiological perspective, from in vitro studies to in vivo assays using oxidative biomarkers. From a chemical viewpoint, many studies have demonstrated how a pigment’s structure influences its antioxidant response and the underlying mechanisms. The major outcome is that this knowledge is essential for interpreting new data in a metabolic networks context in the search for more direct applications to health. A promising era is coming where the term “antioxidant” is understood in terms of its broadest significance.

## 1. Introduction

The term “antioxidant” has various different definitions; the concept has been subject to a continuous evolution as research advances [1,2]. For example, the original chemical definition of antioxidant, “any substance that, when present at low concentrations compared to that of an oxidizable substrate, significantly delays or inhibits oxidation of that substrate” [3], has now been overtaken by a biological perspective, i.e., “natural or synthetic substances that may prevent or delay oxidative cell damage caused by physiological oxidants having distinctly positive reduction potentials, covering reactive oxygen species (ROS)/reactive nitrogen species (RNS) and free radicals” [4]. One main reason for this gradual change from chemistry to biology is the progressive and emerging evidence that supports the involvement of oxidative stress in the development of various diseases like diabetes mellitus, cancer, Alzheimer’s disease, etc.

The differences in how the terms “activity” and “capacity” are applied in the context of antioxidants should be considered. Antioxidant activity is the constant rate of the reaction between an antioxidant and reactive species (radicals and non-radicals); thus, activity correlates with the interaction of that pair of reactive compounds and the quantitation of the structure–activity relationship. Therefore, antioxidant activity is preferentially measured in model systems (homogeneous solutions), and this approach is useful for establishing mechanism(s) of reaction and ranking the antioxidant activity of a family of compounds. That is, we obtain information concerning the chemistry of the process, but that information is not provided within a biological context. Additional terms can be found in the literature, such as “antioxidant power”, “antioxidant potential”, “antioxidant performance”, “antioxidant status”, and “antioxidant effect” [1].

Chlorophyll and carotenoid levels and variability in the diet can be increased through the regular ingestion of fruits and vegetables as well as via the intake of seaweeds, microalgae, functional drinks, and food supplements, as a consequence of new and healthy trends in food habits. Carotenoids and, to a lesser extent, chlorophylls have been investigated regarding their abilities to decrease the oxidation of other molecules. The objective of the present review was to critically construct an updated compilation of the main findings regarding the capability of chlorophylls and carotenoids to decrease oxidative stress. This review encompassed research conducted at the in vitro (outside of the normal biological context, commonly called test-tube experiments), ex vivo (assays in cells/tissues/organs developed outside of a biological environment for up to 24 hours and with minimal alterations of natural conditions), and in vivo levels (experiments developed using the whole organism).

## 2. Methods Used to Determine the Antioxidant Capacity of Lipophilic Pigments

Many protocols for determining the antioxidant capacity of chlorophylls and carotenoids have been published; however, we review herein the methods most commonly applied to lipophilic pigments. Initially, the methods were classified by considering whether the protocol focused on primary or secondary antioxidants. The term “primary” refers to chain-breaking antioxidants, assessed through hydrogen atom transfer-based assay (HAT), single-electron-transfer-based assay (SET), ROS-scavenging activities, metal chelation, or preventing lipid peroxidation, whereas the term “secondary” refers to preventive antioxidants acting through a neutralization reaction [1]. We have introduced how the protocols correlate within a biological context, where detoxifying enzymes, gene expression, transcription factors, or biomarkers are essential indicators of the in vivo antioxidant capacity. Clinical/biochemical biomarkers are, when properly determined, the most direct in vivo measurement of antioxidant status and consequently the most straightforward method with which to determine the impact of an ingested target compound in the health status of a subject. Despite the fact that in vivo results are required to make any health claims (see Section 2.4), there is an open debate in the scientific community regarding the relative significance of each methodology. Several reviews support the utility of total antioxidant capacity (TAC) measurements [5], describing the advantages and limitations of these methods. It has been pointed out that the influence of an antioxidant in health could be independent of the direct effect on human cells and tissues [6]. The presence of an antioxidant in food could prevent or inhibit the oxidation of other food components. Ultimately, the selection of the methodology used to determine antioxidant activity/capacity will depend on the objective being pursued, being aware of the major drawbacks of the selected assay to avoid obtaining inaccurate conclusions. Although numerous classifications have been proposed, we followed an integrated approach [1], starting from pioneer works [7,8,9,10,11,12] and continuing to more recent biochemical assays.

The selection of a suitable protocol depends on factors such as the polarity and structure of the compound, the characteristics of the medium in which the determination is being performed, the feasibility, etc., which are summarized in Table 1. It is important to highlight the case of antioxidant measurements in water/lipid systems that use detergents to produce micellar dispersions of the lipophilic pigments. Some pigments are ineffectively incorporated into micelles under some experimental conditions, and the location of the pigment (in the core or the shield of the aggregates) produces a different result in the radical-scavenging process. Some of the differences attributed to the structural arrangements of pigments and observed in homogeneous systems disappear in heterogeneous systems, whereas other new factors governing the antioxidant process may occur, promoting different and unexpected behavior [11,12,13,14,15,16,17,18,19].

In addition to the results obtained from this milieu of protocols and applications, other controlling factors have emerged, including the spatial distribution of carotenoids and chlorophylls in molecular aggregates or membranes, the heterogeneous and rigid characteristic of the environment surrounding the molecule, the restriction of molecular mobility in the space, and the co-existence of molecules able to interact in the same frame. That is, antioxidant action in heterogeneous environments is a completely different behavioral system, where the concepts and schemes obtained with basic homogenous chemistry are the starting point for ascertaining the actual antioxidant values of lipophilic pigments and any other antioxidant substance.

We have highlighted the influence of the polar paradox in the interpretation of the results when working with chlorophylls and carotenoids as antioxidants. Both families of pigments are, as a general rule, lipophilic pigments, but chlorophylls can also be considered amphipathic molecules. The term “polar paradox” [20,21] refers to polar antioxidants being more active in lipophilic ambient environments than nonpolar antioxidants and, vice versa, nonpolar antioxidants are more effective in oil-in-water emulsions than polar environments. However, recent studies [22] extended the concept with new bases, proposing theories such as the association colloid hypothesis, the cut-off effect, reduced mobility, internalization, or the self-aggregation hypothesis. These new theories have increased the number of factors needed to understand and explain the different behaviors of antioxidant compounds, currently at the in vitro level but with practical applications in the near future.

### 2.1. Hydrogen-Atom-Transfer-Based (HAT) Assays

HAT methods determine the capacity of an antioxidant to quench free radicals by donating a hydrogen atom (H), such as oxygen-radical-absorbance capacity (ORAC) or total radical-trapping antioxidant parameter (TRAP) assays. Although these are considered conventional methods, their applicability is restricted to carotenoids, as this protocol has not yet been applied to chlorophylls. In the ORAC test, peroxyl radicals generated by the thermal decomposition of 2,2′-azobis (2-amidinopropane) dihydrochloride react with a dye and decrease the fluorescence of the target compound, such as fluorescein or a phycoerythrin derivative. The antioxidant capacity of a molecule is measured by its ability to prevent loss of the fluorescence signal by neutralizing peroxyl radicals [7], and this method is especially useful for investigating the efficacy of chain-breaking antioxidants. Antioxidant capacity is generally expressed as Trolox equivalents, abbreviated as Trolox-equivalent antioxidant capacity (TEAC). Opposing results have been reported when comparing ORAC and DPPH methods. For example, a given food might exert a high antioxidant activity with the ORAC test but not with the DPPH assay [8,23], and vice versa.

The TRAP protocol, developed by Wayner [24], has been the most widely used method for measuring the total antioxidant capacity of plasma or serum samples in the last decade. However, its main drawback regarding the oxygen electrode end-point is that the electrode does not maintain its stability over the required time [25]. The TRAP method is characterized by the simultaneous presence of a pro-oxidant molecule, an oxidizable substrate (the target fluorescent protein), and the antioxidant fluid. However, TRAP is not appropriate for the direct measurement of the TAC of lipophilic antioxidants, and is consequently not recommended for nonpolar chlorophylls and carotenoids. However, an alternative (luminol−chemiluminescence-based peroxyl-radical-scavenging capacity (LPSC)) using luminol as the endpoint measurement has been successfully applied for carotenoids [26], comparing antioxidant activity values using different methods. The study noted 10-fold lower antioxidant activity with FRAP, TEAC, and DPPH assays than with the LPSC method.

### 2.2. Single-Electron-Transfer-Based Assays 

Single-electron-transfer-based assays (SET assays) are based on the capacity of an antioxidant substance to transfer one electron to reduce metal ions, carbonyls, and radicals [27] through relatively slow reaction kinetics and dependent on pH and solvents [4]. These methods are not frequently applied for measuring chlorophyll and carotenoid antioxidant activity, although some determinations have been developed through the FRAP or cupric-reducing antioxidant capacity (CUPRAC) protocols.

The FRAP methodology considers the ability to reduce the yellow ferric tripyridyltriazine complex (Fe(III)-TPTZ) to a blue ferrous complex (Fe(II)-TPTZ) by the action of electron-donating antioxidants [28]. The resulting blue color, measured spectrophotometrically at 593 nm, is considered to be linearly related to the total reducing capacity of electron-donating antioxidants [29]. The FRAP assay is the only protocol that directly measures antioxidants (or reductants) in a sample, compared to other assays measuring the inhibition of free radicals [30]. However, one of the inconveniences of the FRAP assay is the need for acidic conditions (nonphysiologically low pH value = 3.6) to maintain iron solubility. The consistency between the FRAP and the ORAC assay was assessed by determining the antioxidant status of watercress, wild rocket, and spinach [31], and the study showed that both methods provided comparable results.

The CUPRAC assay involves mixing the antioxidant solution with a copper chloride solution (II), an alcoholic neocuproine solution, and an aqueous buffer of ammonium acetate at pH 7, and the subsequent measurement of the absorbance at 450 nm after 30 min. The CUPRAC reagent is reasonably selective, stable, easily accessible, and sensitive to thiol-type oxidizers, unlike the FRAP procedure. The CUPRAC method provides advantages over the FRAP protocol since the reaction is performed at almost physiological pH, compared to the unrealistically acidic pH of FRAP [32], and copper redox chemistry, compared to ferric ion chemistry, involves faster kinetics. However, this method is not suitable for chlorophyll *b* determinations, as its maximum wavelength absorbance is very close to 450 nm.

### 2.3. Mixed-Mode Assays

In these methods, different mechanisms (HAT, electron transfer (ET), and proton-coupled electron transfer) may be involved in the antioxidant scavenging of a stable radical chromophore [1]. DPPH (uses 2,2-diphenyl-1-picrylhydrazyl as the radical) and ABTS (applies 2, 2′-azino-bis(3-ethylbenz-thiazoline-6-sulfonic acid as the radical) are the most widespread methods used to determine the antioxidant activity of lipophilic pigments.

The DPPH assay is one of the most widely used protocols, [44,45] and it is currently the Association of Official Agricultural Chemists’ (AOAC) official method (standard 2012.04-2012) for the total antioxidant determination in foods and beverages [46]. The DPPH radical is a long-lived organic nitrogen radical in which purple chromogen is reduced by antioxidant/reducing compounds to the corresponding pale yellow hydrazine. The reducing ability of antioxidants towards DPPH can be evaluated by electron-spin resonance or by monitoring the absorbance decrease at 515–528 nm until the absorbance becomes stable in organic media. A modification to the original protocol was proposed to minimize the interference with carotenoids, shifting the monitoring wavelength to 585 nm [39].

ABTS, when oxidized by peroxyl radicals or other oxidants in the presence of H_2_O_2_, generates a metastable radical cation ABTS^•+^, which is intensely colored and can be monitored spectrophotometrically in the range of 600–750 nm. The evaluation is based on the ability of antioxidant molecules to quench the long-lived ABTS radical cation [47], measuring the decrease in absorbance. Normally, the activity is expressed relative to the Trolox standard [48] as TEAC. The method only detects radical scavengers and is insensitive to other antioxidant effects.

### 2.4. Antioxidant Biomarkers Assessed by In Vivo and Ex Vivo Assays

Widespread chemical antioxidant activity/capacity assays such as ORAC [7], TRAP [49], TEAC [50], LPSC [51], and FRAP [28], are relatively easy to apply and inexpensive, but they are limited by their inability to represent the complexity of biological systems. They measure chemical reactions, and these reactions are not directly correlated to in vivo activity, as they cannot account for the bioavailability, stability, tissue retention, or reactivity of the compounds under physiological conditions. Biological systems are much more complex than simple chemical mixtures, and antioxidant compounds may operate via multiple mechanisms [52]. The different efficacies of compounds in the various assays attest to their functional variation. The best measures are obtained from animal models and human studies; however, these are expensive and time-consuming, and unsuitable for initial antioxidant screening of foods and dietary supplements. Cell-culture models provide an approach that is cost-effective, relatively fast, and addresses some issues regarding uptake, distribution, and metabolism. Wolfe et al. [53] developed a quantifiable cellular antioxidant-activity assay (CAA) based on the peroxyl-radical oxidation (by AAPH or H_2_O_2_) of a bioavailable fluorescent probe forming a fluorescent compound (DCF) within the cells. This method has been successfully applied to carotenoid (Section 3.3.3) and chlorophyll (Section 4.3) antioxidant capacity determination.

Alternatively, multiple oxidatively modified biological molecules are applied as biomarkers of oxidative stress, such as reactive oxygen species (ROS) or reactive nitrogen species (RNS). Along this line and as noted below, the determination of lipid peroxidation products is one of the main methods applied in the study of carotenoid oxidation. Biomarkers such as malondialdehyde (MDA) and lipid hydroperoxides (LOOH), as well as antioxidant assays for estimating lipid peroxidation (β-carotene bleaching assay or thiobarbituric-acid-reactive substances (TBARS)), have been successfully used (see below). To provide a general perspective, in vivo studies frequently determine not only the oxidative status of lipids by measuring oxidized low-density lipoproteins (LDL) particles, lipid hydroperoxides, and F2-isoprostanes, but also damage to DNA/RNA using the comet assay and protein oxidation products, e.g., protein carbonyls. Among the nucleic acid bases, guanine is the most susceptible to oxidation, with 8-hydroxyguanosine (8-OHG) and 8-hydroxy-2-deoxyguanosine (8-OHdG) being the main oxidative biomarkers determined. DNA damage is also frequently monitored using the comet assay, which detects alterations such as single-strand breaks. The formation of protein carbonyls determines the level of protein oxidation, being measured by HPLC, MS, or by immunochemical detection. Carbonyl groups can form via direct oxidation of certain amino acids (Lys, Pro, Arg, Thr, and His) or by scission of the peptide backbone [54]. Some of the oxidative biomarkers are easily monitored through UV–visible spectrophotometry, whereas others require HPLC methods for quantification.

The importance of these biochemical/clinical markers is supported by the requirements of governmental and control organizations before their approval of a health claim. For example, the FDA (Food and Drug Administration) regulates specific requirements for nutritional content claims using the term "antioxidant” [55], while the EFSA (European Food Safety Agency) has published guidance for the scientific requirements for health claims related to antioxidants and oxidative damage [56]. In general, the EFSA does not consider in vitro or even human plasma antioxidant determinations to be sufficient, as these do not establish a beneficial physiological effect in humans. On the contrary, EFSA establishes that “it is necessary at least one appropriate marker of oxidative modification on the target molecule assessed in vivo in human studies,” as shown in Table 2, as a direct in vivo measurement for approval. The EFSA also allows using alternative methods, but only in conjunction with any of the “direct in vivo measurements” listed.

## 3. Carotenoids as Antioxidants: From Magic Bullets to Dietary Trends

### 3.1. A Conceivable Basis to Consider Carotenoids as Dietary Antioxidants

As stated in Section 1, the understanding of carotenoids as antioxidant compounds has changed in recent years from just molecules that interact with radical species to biomarkers associated with decreases in different degenerative diseases, including lung, gastrointestinal tract, pancreas, breast, and prostate cancers; cardiovascular disease; and age-related macular degeneration [57,58,59,60,61,62,63]. This change in vision has promoted the study of the molecular mechanisms that allow carotenoids to be involved in such multifactorial processes, with corresponding significant advances in knowledge regarding the appearance of carotenoid metabolites [64,65,66,67], the absorption and transport processes responsible for the accumulation of carotenoids in specific organs and tissues [67,68], and the increasing number of metabolic pathways in which carotenoids can act, resulting in a substantial benefit for health [56,69,70]. However, not all the processes through which carotenoids perform their activities have been clarified or even envisaged, and some are not specifically correlated with antioxidant action (retinoid-dependent signaling, enhancement of gap-junction communication, induction of detoxifying enzymes). Most of these activities could be performed in a non-antioxidant manner; therefore, the association between the terms “carotenoids” and “antioxidants” does not fully represent the capabilities of this family of natural pigments.

**Figure 1:**
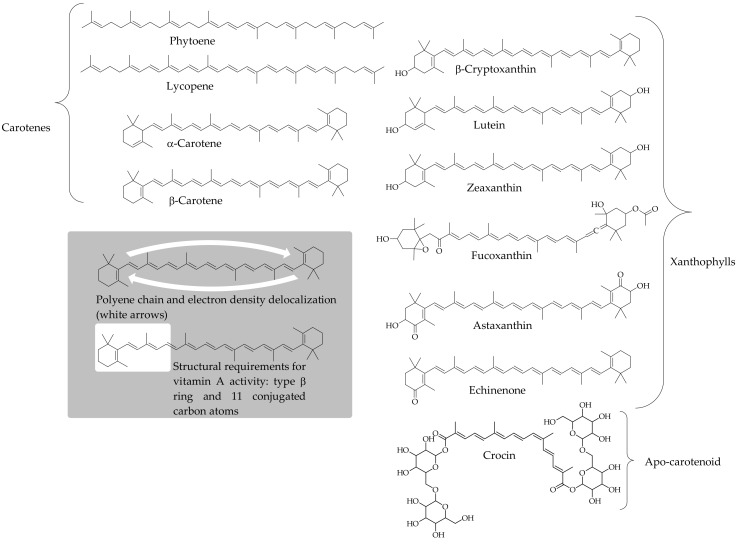
Structure of carotenes and xanthophylls commonly present in dietary sources. The inset represents the conjugated system double-bound through the central carbon atom chain, and the structural requirements that a carotenoid must fulfill to produce vitamin A activity.

Carotenoids are isoprenoid-based molecules distributed in all photosynthetic organisms, and some non-photosynthetic bacteria and fungi. In these organisms, the initial steps of the biosynthetic pathway aim to yield the 40 carbon phytoene intermediate. Once this common precursor is produced, a considerable number of chemical and enzymatic processes (desaturation, cyclization, hydroxylation, epoxidation, cleavage of the polyene chain, etc.) may occur several times and, alternatively combined, can yield a wide range of structural arrangements of carotenoid products [71]. Hence, around 1100 carotenoids have been identified in nature, and they are classified into two groups: carotenes (pure hydrocarbons) and xanthophylls (oxygenated products of carotenes). Figure 1 depicts the structures of representative carotenoids, emphasizing those consumed regularly in the human diet, and denoting the characteristic structural arrangements commonly present in these pigments. Carotenes and xanthophylls can be arranged to form the common C40 structure, although biosynthesis of C30 and C50 carotenoids has been described in bacteria, whereas apo-carotenoids are metabolites resulting from shortening the C40 structure of the parent compound. Carotenoids are characterized by their bright yellow to red colors, although some colorless precursors can contribute to the commonly diverse carotenoid profiles that accumulate in organisms. The attractive colorful natures of these compounds results from an extensive chain of conjugated double bonds (Figure 1), and this structural arrangement is the first interactive center of carotenoids with other molecules in the environment [71]. Animals rely on their diet to incorporate these pigments from natural sources, mainly fruits and vegetables, algae, eggs, and fish [72]. The rich variety of carotenoids is far from accessible to us. Our dietary patterns provide us with regular access to 40 carotenoids [73], although this number could significantly increase via the rising inclusion of microalgae in our diet [74]. However, the carotenoid profile in human tissues is mostly comprised of α-carotene, β-carotene, β-cryptoxanthin, lycopene, lutein, and zeaxanthin [75]. The analysis of human plasma has shown that it contains these and a few other carotenoids, depending on the dietary habits of the study population, whereas even a small number of the pigments mentioned above can accumulate in other tissues, such as lutein and zeaxanthin in the macula lutea, lycopene in the prostate, or xanthophyll esters in human milk [76,77,78].

The function of carotenoids in a variety of animal species, including humans, is to act as the major precursors of vitamin A, necessary for vision, growth, cell differentiation, and other physiological processes [79]. Only carotenoids with at least one unsubstituted type β-ring and polyene chain containing 11 carbon atoms (Figure 1) belong to the provitamin A category (β-carotene and its isomers, α- and β-cryptoxanthin, γ-carotene, mutatochrome, β-zeacarotene, and β-apo-8′-carotenal are main dietary carotenoid contributors to provitamin A activity). This function of carotenoids is key for people that depend on fruits and vegetables to meet their daily required amounts of vitamin A both in developed and developing countries [80,81]. The significance of this single function of carotenoids in human nutrition does not overshadow some functions performed by carotenoids in photosynthetic organisms that could also somehow be performed in animals. 

**Figure 2:**
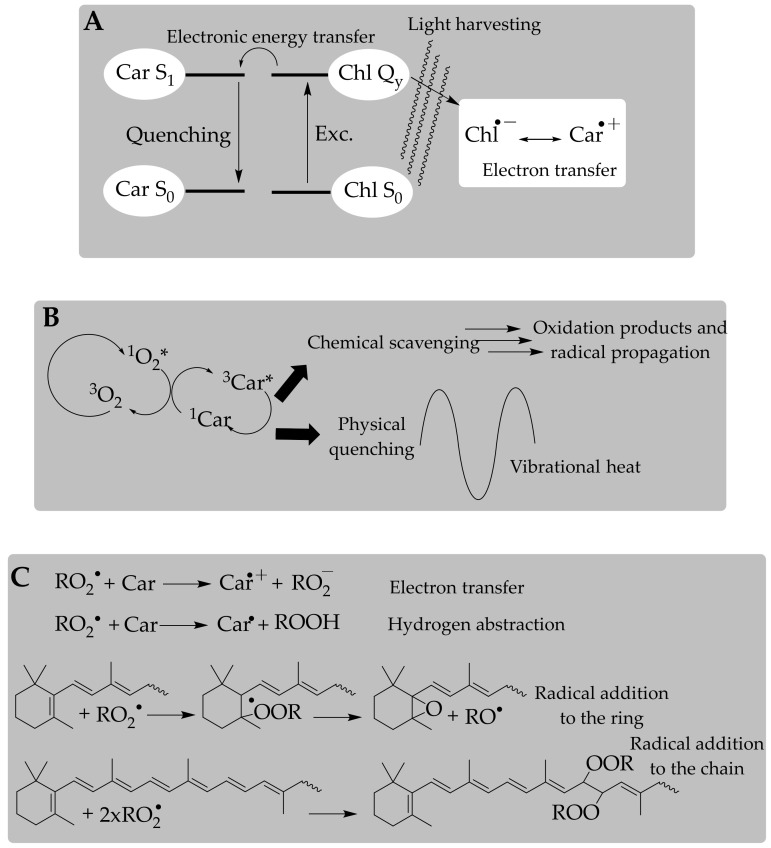
Mechanisms of the antioxidant action exerted by carotenoid pigments in different processes. The electron transfer from the characteristic conjugated polyenic chain of carotenoids to chlorophylls (**A**). Physical quenching of singlet oxygen (**B**). Electron transfer, hydrogen abstraction, and radical addition in the antioxidant activity against peroxyl radicals (**C**).

Carotenoids are essential in photosynthesis for the light-harvesting process and protection against photo-oxidative damage, so we expect carotenoids to function as antioxidants in any organism independently of whether it is photosynthetic. The electron transfer from the characteristic conjugated polyenic chain of carotenoids to the photosystem reaction centers produces an excited state that is essentially dependent on the structural characteristics of the end-groups (Figure 2A). This function in light-harvesting complexes could be exported to other environments, including both cellular and organelle membranes, adipose tissue, and circulating plasmatic lipoproteins, where carotenoids from the diet are accumulated in animals. However, there are two issues. First, the antioxidant function of carotenoids in photosynthetic organisms acquires the status of action or activity in non-photosynthetic species, but carotenoids may behave as pro-oxidants under certain circumstances [82], a feature that has not been described in the natural environment of these pigments so far. In line with this idea, we stress that the natural antioxidant function does not necessarily mean an effective in vivo antioxidant action; unexpected (deleterious) activities could occur.

The second step was establishing their mode of action, which was promptly formalized [83]. Authors determined carotenoids’ high ability to quench singlet oxygen, which is a physical phenomenon through which the excitation energy of singlet oxygen is transferred to the carotenoid molecule and then harmlessly released to the environment, so that the carotenoid finally reaches its original ground state (Figure 2B). This quenching ability is limited and, after several quenching cycles, the carotenoid is oxidized [84]. Carotenoids are nevertheless recognized as potent quenchers of singlet oxygen and the triplet state of porphyrin structures (chlorophylls and protoporphyrin); this was potentially the basis for searching for additional modes of action toward other oxygen free radicals (superoxide anion radical, and hydroxyl and peroxyl radicals), measuring their antioxidant effectivity, and investigating the possible effect of these pigments in the progression of diseases involving radical species [85,86,87,88]. The mechanisms and kinetics of these antioxidant process, generally denoted as radical-scavenging chemistry (Figure 2C), have been mainly measured in vitro, ex vivo, and in cell culture models, whereas representative animal studies and clinical trials that appropriately evaluate the in vivo antioxidant effect of carotenoids are limited. 

### 3.2. Antioxidant Activity of Carotenoids: In Vitro Approach of a Chemical Process

Carotenoids are lipophilic antioxidants, with a few exceptions such as crocin and its derivatives which are water-soluble compounds (Figure 1), and they react via different mechanisms, e.g., electron transfer, hydrogen abstraction/reduction, and formation of carotenoid–radical adducts, depending on the redox potential of the radical species and the structural arrangement of the carotenoid.

The electron-transfer mechanism (Figure 2C) involves high-redox-potential radical species [88], and the product of the process is a carotenoid radical cation that decays through dismutation by second-order kinetics [89]. Carotenoid radical cations are long-lived intermediate products and they have a strong oxidizing potential, easily damaging biomolecules (tyrosine, cysteine) [90]. Comparative studies of the antioxidant activities of different carotenoids have reported relative one-electron oxidation potentials that show how xanthophyll radical cations are regenerated to their parent state by carotenes [91]. This repair capacity is also provided by other antioxidants (ascorbic acid, α-tocopherol) to carotenoid radical cations [90,92]. The hydrogen abstraction mechanism is a consequence of reduction processes that are correlated with the redox potential of the radical species and with the structural arrangement of the carotenoid. Hence, carotenoids with hydrogen(s) in the allylic position to a double bond react with radical species to yield a resonance-stabilized radical that may continue the radical propagation chain (Figure 2C). This reduction process was extensively summarized in the reviews published by Britton [93] and Edge et al. [94].

These mechanisms were analyzed using a group of techniques specifically designed to monitor fast reactions (pulse and laser flash photolysis, electron-paramagnetic resonance with spin-trapping capture, and thermal decomposition). The production of carotenoid–radical adducts via radical addition is in some ways the most interesting mechanism, because it involves radical species resulting from the lipid peroxidation process (Figure 2C). In this case, the translation of the antioxidant activity measurement from solutions to molecular aggregates and membranes is logical. The key step is the addition of the radical species to the carotenoid molecule, yielding a radical intermediate through which an unpaired electron is effectively delocalized through the polyene chain [82]. Here is where the structural arrangements of the end-groups play a significant role in the fate of the carotenoid–radical adduct, as the former’s characteristics could contribute to enhancing the resonance-stabilized process of the latter species, and consequently its reactivity [95,96]. Methodologies based on the reaction chain of the lipid autoxidation process are the basis for establishing protocols to determine antioxidant activity [97,98].

As stated above, the structural arrangements of carotenoids point to the fate of the carotenoid–radical intermediates, but experimental conditions also contribute to the prevalent type of scavenging mechanism involved and the resulting observed effect. Burton and Ingold [82] showed that β-carotene is an effective antioxidant at low oxygen tensions (<150 Torr), but when higher values are experimentally set up, the behavior switches to a pro-oxidant activity. This indicates that a close interaction exists between the carotenoid structure and oxygen, and that the carotenoid–radical intermediates propagate the effect of the starting radical species under those experimental conditions. However, oxygen tensions in normal physiological conditions are in the range of 145–165 Torr [99] for the lungs and drop to 15 Torr or even less in other tissues [100], so the possible pro-oxidant effect of carotenoids for in vivo systems should be attributed to factors other than oxygen tension.

### 3.3. Antioxidant Capacity of Carotenoids: A Further Step to Estimate the In Vivo Antioxidant Action

Antioxidant capacity is defined as the number of radical species that are removed or neutralized from the environment by an antioxidant substance. That capacity action can be tested in homogeneous environments, as are used to estimate the antioxidant activity. Thus, several protocols can be used to assess how carotenoids scavenge radical species, as reviewed previously, mainly using the oxygen-radical-absorbance capacity (ORAC) [7], total radical-trapping antioxidant parameter (TRAP)-related protocols [24,49,101], ABTS assays [47], and DPPH testing [45]. The chemistry of these methods is expected to predict the antioxidant capacity in in vivo systems with a relatively high-throughput screening ability.

However, some improvements in the design of antioxidant capacity measurements should be introduced if we want to increase the reliability of the data and support the correlation of experimental predictions with in vivo scenarios. The first notion to consider is the fact that carotenoids are transported in lipoproteins and accumulate in the membranes of cells and organelles of tissues. Therefore, experimental procedures with that aim should be combined into one protocol to reproduce the natural surroundings in which carotenoids accumulate, i.e., membranes or molecular aggregates of lipophilic compounds (lipoprotein particles), with one or various protocols used to measure the antioxidant capacity in this mimicked system. Three approaches are commonly applied to build carotenoid-rich domains: preparing artificial membranes or liposomal aqueous suspensions, isolating biological samples (plasma) from a human or animal diet supplemented with antioxidants, or using cell-culture models, which have some other issues in addition to the antioxidant capacity, such as uptake, distribution, and metabolism of the tested antioxidant. Each of these approaches is complemented by a protocol that induces the appearance of one or several radical species, which are scavenged by the membrane antioxidant(s) being tested.

#### 3.3.1. Measurement of the Antioxidant Capacity in Liposomes

The liposome is the simplest approach for studying biological membranes, and is still a significant tool used to manage issues regarding the biochemistry of membrane components. Liposome preparation is easy, taking advantage of the spontaneous aggregation of phospholipids in aqueous environments as closed membrane systems [102]. These membranes are relatively stable, produced with an exactly known and “made-to-order” lipid composition, and may reproduce the fate of membrane lipids in terms of the interfacial phenomena occurring inside and outside of the membrane structure.

Table 3 lists some relevant studies that focused on the antioxidant capacity of carotenoids in liposomes, describing the main features of the experimental protocols and the observed effects. Most of the studies reported a positive correlation between the presence of carotenoids in the membrane system and a lower incidence of the radical-induction process. Additionally, several of the studies included the appearance of synergistic effects in the antioxidant capacity. The method may provide an efficiency ranking of the antioxidant capacity, but significant divergences may arise from the lack of standardization of the protocols (liposomal membrane preparation and radical species strategy). Thus, the following should be considered: diameter and number of lipid bilayers (small unilamellar vesicles, large unilamellar vesicles, and multilamellar vesicles); ratio among lipid contents used to build the artificial membranes; preparation parameters applied; and the presence of membrane antioxidants that may influence membrane properties, such as fluidity, polarity, thermostability, and distribution of lipid categories. All these variables make comparison between studies less than straightforward.

Some of the antioxidant studies of carotenoids performed using the liposome approach revealed properties related to the orientation of these pigments in biological membranes and their ability to suppress radical penetration of oxygen into such membranes. Another interesting lesson learned from this kind of procedure is that the cooperative action between different antioxidant categories produces a synergistic effect on the capacity, a significant result regarding the design of intervention trials and clinical studies. 

#### 3.3.2. Measurement of the Antioxidant Capacity in LDLs

The ex vivo oxidation of LDL is a method used to evaluate the effect of dietary supplements or food antioxidants on the oxidizability of lipoprotein particles using a fusion of test-tube protocols with in vivo studies, so that the pros and cons of both approaches are joined in a single procedure. The experimental animals or human volunteers participating in such studies follow a specific diet, and the lipoprotein fraction is enriched in these pigments. The first advantage of this method is that the natural particulate matter in which carotenoids are packaged and circulate through the body is accessible. The efficacy of the metabolic processes required to reach the targeted accumulation of carotenoids in lipoproteins is affected by several factors, so the heterogeneity of the biological samples could be high and other antioxidants in addition to carotenoids could be targeted to the lipoprotein fraction.

Another issue to consider is the protocol applied to induce the oxidation of lipids in the isolated lipoprotein fraction and the corresponding selected biomarker for quantification. Here, as detailed in the previous section, there are several options that follow the increase in the molecules that are generated in reaction with radical species, such as the products from lipid peroxidation, including aldehydes (malondialdehyde, 4-hydroxy-2-nonenal), conjugated dienes, and lipid hydroperoxides, or products arising from the oxidation of lipoproteins, including carbonyl groups, and advanced glycation end-products. In addition, antioxidant-capacity protocols such as FRAP or ABTS are directly applied to the isolated lipoprotein fraction. Finally, the antioxidant status of the individual before and after the intervention study can be estimated via the measurement of urine or plasma biomarkers (prostaglandin-like compounds and derivatives, DNA-damage products, reduced/oxidized glutathione (GSH/GSSG) ratio, etc.).

Table 4 shows some of representative human studies that applied the ex vivo oxidation of LDL method to carotenoids, indicating the characteristics of the intervention group (healthy subjects or population at risk of oxidative-stress-related pathologies), diet, the analyzed biomarkers, and the outlined conclusions. Most of the studies showed that after enrichment of LDL with carotenoid(s), they were less prone to oxidation, whereas no effect or even a deleterious action was the effect observed in other studies. To clearly ascertain the quality of such evidence, various critical issues should be considered, such as the design of the intervention trial (randomization, placebo-controlled, blinding), the characteristics of the study population (healthy, smokers, patients), the intervention time and dosage strategy (single-compound or cocktail of antioxidants as supplements, food extracts, or foods), and the number of biomarkers used to control the antioxidant capacity. Hence, those studies where the intervention was aimed to be a preventive therapy provided positive results. In contrast, when therapeutic measurement was the focus, the results tended to be unclear for one or several biomarkers, and the conclusions were inconsistent. Another issue is that in the studies where a mixture of antioxidants was used, including different carotenoids as well as tocopherols, phenols, or ascorbic acid, the tendency was towards a positive relationship between increased levels of antioxidants in LDL and resistance to oxidizability. The same trend was observed for studies with foods instead of supplements, or when the content of antioxidants supplied to the volunteers with the latter products did not reach supraphysiologic levels. Interpreting whether these results should be attributed to an increase in carotenoid levels of LDL or to other components of the diet is difficult.

#### 3.3.3. Measurement of the Antioxidant Capacity in Cell Models: Carotenoid Oxidation Products

Cell-culture models were the previous proof of concept for in vivo studies, and allowed researchers to ascertain the behavior of antioxidant in more complex situations. As such, the antioxidant effect of carotenoids in cellular models encompasses the protective effect on membrane lipids with the (indirect) reduction in oxidative stress of other significant biomolecules, including DNA [123]; the modulation of cellular responses to inflammation mediated by nuclear receptors [124,125,126]; the stimulatory effects on a cell-to-cell communication via gap junctions and cell growth [127,128]; and the activity of oxygenases in mitochondrial membranes as a source of carotenoid breakdown products [129,130,131]. Most studies performed with cell-culture models were established with cell lines representing tissues where carotenoids are transported, accumulated, or metabolized, such as intestinal epithelium, macrophages, adipocytes, human dermal fibroblasts, keratinocytes, and retinal pigment epithelium; erythrocytes, retinal neurons, and other cell lines have also been applied. Table 5 includes representative studies conducted with cell-culture models. Significantly, most of the studies reported positive results, pointing to a synergistic effect with other membrane antioxidants and outlining the contribution of carotenoid breakdown products to possible deleterious effects on the expected antioxidant behavior. Studies in cell cultures provided the basis of our knowledge of the non-antioxidant actions of carotenoids and other membrane antioxidants, as well as pointing to plausible modes of actions in completely different arenas, such as experimental animal models and clinical studies and analyses of epidemiological data [132]. Thus, studying the antioxidant-responsive elements that mediate the transcriptional control of the expression of Phase II enzymes (see below), the identification of carotenoid breakdown products, and the general response to inflammation are research issues currently outstanding, and should be directly evaluated at the in vivo level. To establish the quality of evidence, various critical issues and study limitations should be considered, such as the dosage strategy (physiological or supraphysiological dose, application of the antioxidant supplement to the cell culture in organic solvents, micelles, or lipoproteins), the intervention time, and the number of cellular biomarkers used to control the antioxidant capacity.

## 4. Antioxidant Capacity of Chlorophylls

This section summarizes the scientific evidence that supports the antioxidant properties of different chlorophyll compounds, emphasizing issues that arise in the diverse environments where chlorophylls could exert this role. However, the one exception to this attribute is the reported pro-oxidant activity of chlorophylls in lipophilic environments under light conditions [146], where positive results have also been obtained [147]. With this exception, previous reviews described different aspects of the antioxidant capacity of chlorophylls [148,149,150,151,152], but we compiled the research formulated from diverse perspectives starting with the fundamental studies of the antioxidant capacity of chlorophyll standards, through the search of new sources of dietary antioxidants, to the ongoing studies of the in vivo chlorophyll antioxidant actions. However, in contrast to the carotenoid studies, knowledge is limited regarding the yield of chlorophyll metabolites, their absorption and transportation processes, their metabolic pathways, and their precise oxidation mechanisms. At the in vitro level, only few researchers have studied the stability of chlorophylls during digestion [153,154,155,156,157] and subsequent absorption through intestinal cells [153,154,155,157,158]. The major outcome is that chlorophylls *a* and *b* are transformed into their corresponding pheophorbides and pheophytins and are absorbed at similar rates as carotenoids. A further step has been to show that pheophorbide *a* is transported at the intestinal level by a protein-mediated mechanism, with scavenger receptor class B type 1 (SR-BI) being a plausible transporter [159]. These results have been confirmed at the in vivo level, using mice as the experimental model, showing a preferential accumulation of pheophorbide in the liver along with multiple other chlorophyll compounds [159]. However, in humans, a trial was developed with copper chlorophylls, identifying copper chlorophyll e4 as the major component in serum [160]. In spite of this promising result, knowledge regarding chlorophyll assimilation is still scarce. In parallel, the number of studies considering the in vitro oxidation activities of chlorophylls is low compared to those examining carotenoids. However, a new and promising set of assays showing the health-promoting activities of chlorophylls has promoted the development of studies dealing with their in vivo antioxidant actions. This research area provides opportunities for unraveling chlorophylls’ actions in animal metabolism.

### 4.1. Different Chlorophyll Standards

**Figure 3:**
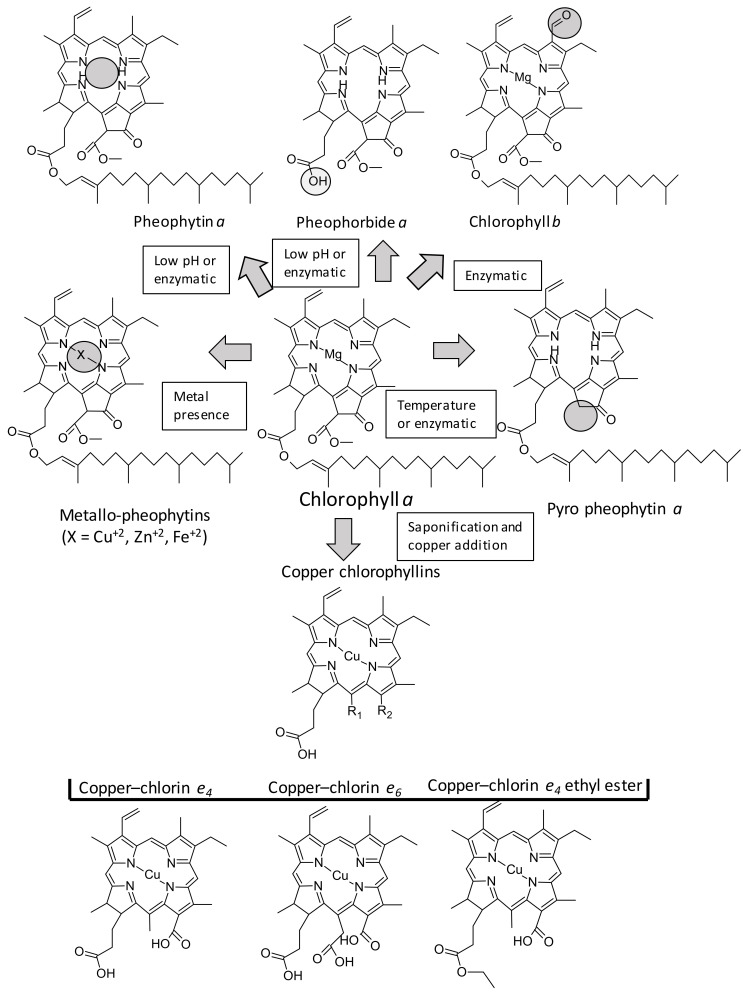
Chlorophyll structures cited in this paper.

Chlorophylls are cyclic tetrapyrroles carrying a characteristic isocyclic five-membered ring (Figure 3), which show different functional groups in constrained positions, yielding more than 100 different structures present in nature. However, the antioxidant properties of chlorophylls have been studied with the most common chlorophyll derivatives, which are depicted in Figure 3, highlighting the main substituted positions (C7, C13^2^, C17^3^, and the central metal). Starting from chlorophyll *a*, the replacement of the methyl group by an aldehyde at C7 forms *b*-series chlorophylls; the central magnesium can be exchanged by hydrogen (pheophytins) or metal ions such as copper, zinc, or iron (generating the metallo-chlorophylls); the additional desesterification of the phytyl group (C_20_H_40_) at C17^3^ creates pheophorbides; and the loss of the carboxymethyl group at C13^2^ generates pyro-derivatives. The structure and configuration of chlorophylls affect their antioxidant activity, and hence different studies have addressed this question. The first step was to elucidate the differences between *a* and *b* series, and contradictory results were reported for *a* and *b* chlorophylls (Table 6). For example, some reports [149,161,162] found that *b*-series chlorophyll compounds exhibit higher antioxidant activity than *a*-series ones, suggesting an unknown role for the aldehyde group at C7 in the antioxidant ability. Conversely, with similar tests, assays from Schwartz and colleagues [148] showed chlorophyll *a* to be three times more effective of a radical quencher than chlorophyll *b*, in agreement with previous results reported with the ferric-nitrilotriacetate-induced singlet oxygen lipid peroxidation assay [163] or by the CUPRAC assay [37].

Unlike the influence of the *a*/*b*-series factor in the antioxidant action, and independent of the method applied, consensus exists on the positive role of the central metal [148,149,164,165]. Metal-free chlorophyll derivatives (pheophytins, pheophorbides, etc.) exhibit significantly lower antiradical capacity than metallo-derivatives, with the presence of copper being more favorable than zinc or magnesium. It has been hypothesized [148,157] that the presence of the metal could increase the electron density at the center of the skeleton, thereby enhancing the ability of the conjugated porphyrin backbone to donate electrons. An additional explanation is based on the ability of the π-cation radical in the porphyrin structure to induce the donation of electrons from the porphyrin structure in order to break the propagation of the radical chain process [166]. However, some authors, finding a higher antioxidant capacity in pheophorbides than in metallo-chlorophylls, speculated that nonmetal chlorophylls might exert their antioxidative capacity via their inherent ion-chelation capacity in addition to the porphyrin stabilization of ROS [162].

Along this line, the wrongly denominated “copper chlorophyllins” (or even, sodium copper chlorophyllins (SCCs)) demand special attention. These compounds constitute the hydrophilic green food colorant (E-141ii) commonly used in ice cream, candies, cookies, and desserts [167]. They are produced from native chlorophylls after a solvent–saponification reaction and copper addition, producing a highly stable food colorant. This commercial product is formed by a diverse array of copper chlorophyll structures characterized by the lack of phytol at C17^3^; they mostly present the ring V-opened with multiple functional groups (R_1_ and R_2_, Figure 3). Apart from their attractive coloring properties, this set of chlorophyll derivatives consistently exhibit the highest antioxidant activities, as much as three to five times higher compared with their un-coppered counterparts [148]. The same outcome was obtained through different in vitro analytical methods, including DPPH [168], ABTS [148], and ß-carotene test, based on the quantification of β-carotene decoloration due to the radicals generated during the oxidation of fatty acids [149]. This capacity has been positively determined through the noted in vivo assays, such as the comet protocol or biomarkers such as MDA, in incubated human blood cells oxidized with γ-radiation [169], demonstrating the oxidative protection of copper chlorophyllins.

### 4.2. Antioxidant Activity of Chlorophyll Extracts from Different Sources

The knowledge of the antioxidant competence of purified molecules constitutes the first and fundamental step that should be pursued. However, we must remember that the real scenarios in which these molecules exert their actions are intricate environments with multiple interactions. Foods should be considered complex matrices, where the isolation of each compound could be challenging despite synergies or antagonisms [2] with other compounds, effects that are not observed when the antioxidant activity is measured using a pure standard. Hence, analyzing the antioxidant capacity of chlorophylls from another perspective would involve studying complete foods, organisms, or extracts. However, for this approach, a battery of antioxidant assays based on different reaction mechanisms should be applied instead of a single assay as the best strategy to obtain accurate results. In this sense, these main topics should be considered when analyzing food products [2], selecting a method, or evaluating the obtained results: the working pH, a wide range of hydro-lipophilicity, kinetics of the main antioxidants, the base color of the food, and the relative amount of proteins.

The seaweed industry has an annual value of USD $5.5–6 billion, with 90% of the production being for human food products, mainly for their high nutritional value. Chlorophylls are among the secondary metabolites responsible for the health benefits of seaweed consumption. For example, five brown species of seaweed are able to scavenge peroxyl radicals due to their chlorophyll *a* content, which shows a synergistic effect with vitamin E [174]. However, Chlorophyceae (green seaweeds) are the most investigated species [175]. *Enteromorpha prolifera* has excellent antioxidant properties with strong DPPH-radical-scavenging activity, reducing power, and hydroxyl-radical-scavenging activity due to their pheophorbide *a* content [176]. 

Spirulina has recently been approved as a food, opening the door to new food formulations enriched with different microalgae. For example, the dried biomasses of four microalgae strains, *Arthrospira platensis*, *Chlorella vulgaris*, *Tetraselmis suecica*, and *Phaeodactylum tricornutum,* were used to prepare wheat crackers [177]. The authors reported that higher amounts of microalgae increased the antioxidant capacity measured via the DPPH method. Accordingly, pasta enriched with microencapsulated Spirulina increased the antioxidant potential after cooking [178]. Using a different method, namely the peroxyl-radical-scavenging assay, the chlorophyll fraction in *Phormidium autumnale* was found to be responsible for a high antioxidant activity, around 200 times more potent than α-tocopherol [171].

Another line of research is the analysis of plant extracts to identify rich sources of edible antioxidants. For example, the chlorophyll content (*a* and *b*) of stem amaranth (*Amaranthus lividus*) leaves has a significant positive correlation with the total antioxidant content (measured through DPPH and ABTS methods) [179], indicating this leafy vegetable as a potential source of antioxidants in the human diet. Several medicinal plants show high antioxidant capacity, some of them at the same level as ascorbic acid [180]. Elimination of chlorophylls from different cultivars of jalapeño and serrano peppers drastically reduced the antioxidant activity estimated by the DPPH-scavenging assay and measured by electron-paramagnetic resonance (EPR) spectroscopy [181]. This antioxidant action was also observed in vivo. Chlorophyll extracts from *Sauropus androgynous* (L.) leaves intraperitoneally administered to Wistar rats for 14 days were able to protect the liver and kidney from the oxidative stress caused by sodium nitrate [182].

Despite their functional properties, the use of chlorophyll as a functional ingredient has been limited to some extent due to its chemical instability. To address this, a new approach was constructed, using different microencapsulation techniques as a strategy to retard chlorophyll oxidation, enhance water solubility, improve stability, and extend shelf life. For example, kale chlorophylls were microencapsulated in isolated whey protein, which increased their antioxidant activity 20% as assessed through the DPPH method [183].

### 4.3. In Vivo Antioxidant Activity of Chlorophylls

Multiple biological functions have been reported for chlorophylls. The most known is probably the ability to trap mutagens based on the planar structure of chlorophylls, which allows the availability of deleterious compounds in the cell to be reduced [170]. Strictly related to their antioxidant capabilities, two main mechanisms can be described: their direct free-radical-scavenging activity and the metabolic activation of detoxification pathways.

#### 4.3.1. In Vivo Free-Radical-Scavenging Properties of Chlorophylls

**Figure 4:**
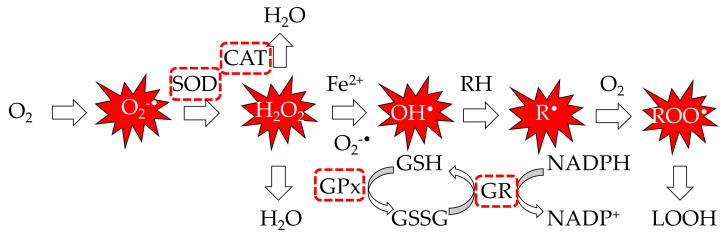
Reactions and enzymes involved in reactive oxygen species (ROS) generation and propagation analyzed in chlorophyll antioxidant studies: superoxide (O2·−), hydroxyl (OH·), peroxyl (RO_2_·), and hydrogen peroxide (H_2_O_2_). Antioxidant enzymes: SOD: superoxide dismutase, CAT: catalase, GPx: glutathione peroxidase, GR: glutathione reductase, GSH/GSSG: reduced/oxidized glutathione.

Regarding the first mechanism, Figure 4 describes the process of formation and propagation of different ROS (superoxide (O2·−), hydroxyl (OH·), peroxyl (RO2·), alkoxyl (RO·), hydroperoxyl (HO2·), and nonradical species such as hydrogen peroxide (H_2_O_2_) and singlet oxygen (^1^O_2_). The common experimental approach in these studies is the analysis of different tissues from experimental animals (mice, rats, etc.) subjected to diets rich in chlorophyllin (probably copper chlorophyllins). The results demonstrated the capacity of chlorophylls to reduce the general ROS levels at the in vitro level [184,185,186,187]. As stated above, excess ROS generation in cells induces damage in lipids, proteins, and DNA, making oxidized biomolecules perfect biomarkers of in vivo oxidative status. The pioneer works on the inhibition of lipid peroxidation by copper chlorophylls were developed by Sato’s group [168,188]. Later, this effect was reported in numerous tissues, normally through the formation of lipid hydroperoxides [183], but also through the TBARS method: in lymphocytes in a concentration-dependent manner up to 75% [185], in kidney and heart tissues at the same level as ascorbic acid [189], and in the liver [190,191,192], brain [187], and serum [182].

ROS also react with DNA, mainly yielding base radicals through the formation of double bonds and occasionally producing deoxyribose radicals by abstracting hydrogen atoms [193]. In any case, both situations can result in strand breakage, which has been used as an oxidative biomarker to measure the antioxidant activity of chlorophylls. For example, using an in vitro plasmid DNA system, the rate constant for the reaction of OH· and 2-ROO [194] with chlorophyllin has been calculated, resulting in similar figures to other well-known antioxidants (such as GSH) and pointing to the effective role of chlorophyllin as an oxidative protector of DNA. At the in vitro level, different non-copper chlorophyll compounds (chlorophyllide *a* and *b* and pheophorbide *a* and *b*) are able to reduce single-strand DNA breaks and levels of 8-OHdG in human lymphocytes, although pheophorbide derivatives displayed higher scavenging capacities than others. These indicators have also been determined at the in vivo level. Male rats fed a chlorophyllin diet showed lower levels of liver microsomal MDA, DNA fragmentation, restriction-fragment-length polymorphism (RFLP), and 8-OHdG concentration in comparison with a control diet [195]. As a new analytical approach, immunohistochemical techniques have been developed to allow the measurement of DNA oxidative indexes, for example to prove the capacity of chlorophyllins to reduce the formation of 8-OHdG in cancer-induced hamsters [196].

Finally, the main biomarker of oxidative stress in proteins is the formation of carbonyl (CO) groups (aldehydes and ketones), especially on Pro, Arg, Lys, and Thr residues [197]. Measuring this oxidative biomarker, copper chlorophyllin has shown its ability to decrease the level of protein carbonyls in kidney and heart tissues at rates similar to those of ascorbic acid [190] in liver tissues [184,188,192]. An even more marked effect was observed when *Caenorhabditis elegans* consumed a pheophorbide-rich diet, reducing the carbonyl groups up to 80% in comparison with a control group [198].

Concomitant with this capacity to reduce the level of oxidation in the main biomolecules, chlorophyll derivatives are effective enhancers of the activity of the main enzymes implicated in the antioxidant machinery at the cellular level (Figure 4). For example, mice fed 1% chlorophyllin for three days in drinking water were euthanized and mitochondrial isolates from the liver were exposed to γ-rays to induce oxidative stress. Chlorophyll-treated rats were able to restore the SOD activity depressed by radiation to a greater extent in oxic compared to anoxic conditions [184]. 2,2′-Azobis(2-methylpropionamidine) dihydrochloride (AAPH)-oxidative-stress-induced mice were injected ex vivo with 100–400 mg/g body weight (b.w.) copper chlorophyllin, and the corresponding lymphocyte lysates showed increased catalase and GPx activities in comparison to control samples [185]. Hamsters with 7,12-Dimethylbenz[a]anthracene (DMBA)-induced cancer provided with a 14 week chlorophyllin-supplemented diet showed increased expression (assessed by semiquantitative RT-PCR) of SOD, catalase, and GPx enzymes, in agreement with their signals in the corresponding immunoblot analysis [197]. Diabetic mice with oxidative stress induced by streptozotocine showed activation of detoxification pathways (CuZnSOD, MnSOD, CAT, GPx, and GR) in response to alternative and intraperitoneal doses of 50 mg/kg b.w. of copper chlorophyll for 28 days in kidney, heart [190], and liver tissues [192]. More recently, diabetic Wistar rats were in situ perfused with chlorophyll-based extract or chlorophyll *a* standard and subjected to light irradiance to induce oxidative stress. The treatment group showed lower hepatic oxidative stress, and lower expression and activity of CuZnSOD, MnSOD, CAT, and superoxide dismutase in liver tissues [188]. The next step would be to investigate the exact mechanisms by which chlorophylls activate these antioxidant enzymes.

#### 4.3.2. In Vivo Activation of Detoxification Pathways by Chlorophylls

**Figure 5:**
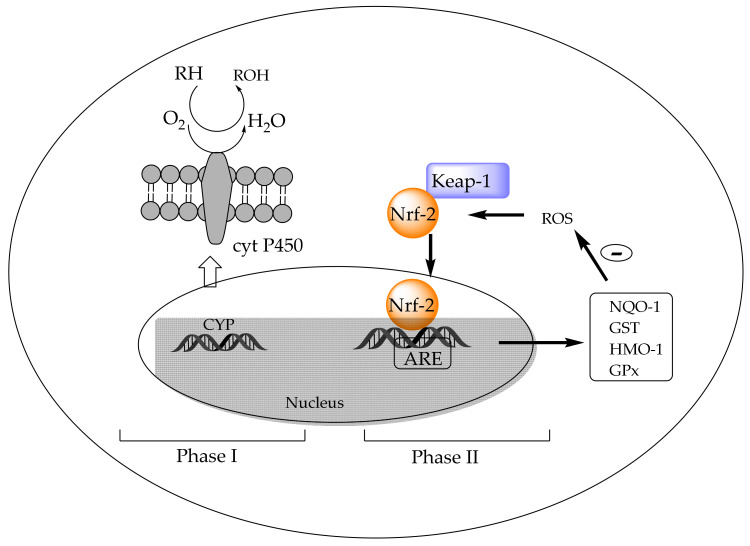
Detoxifying metabolism: Phase I and Phase II. *CYP*: cytochrome P450 monooxygenase, ARE genes: antioxidant-response elements; Nrf-2: nuclear factor erythroid 2 related factor 2; Keap-1: Kelch-like epichlorochydrin-associated protein 1; NADPH quinine oxidoreductase 1: NQO-1; glutathione S-transferase: GST; heme oxygenase 1: HMO-1; glutathione peroxidase: GPX.

In addition to antioxidant enzymes, living cells develop programmed reactions to reduce the potential injury caused by xenobiotics through specific metabolism and later excretion. The group of enzymes involved is known as drug-metabolizing enzymes (DMEs), and their sequential function allows them to be categorized as Phase I, II, and III enzymes. Phase I enzymes oxidize drugs or xenobiotics, for example, cytochrome P450 monooxygenase (CYP), the expression of which is governed by several nuclear receptors. Phase II enzymes conjugate products of Phase I reactions, such as glutathione peroxidase (GPX), glutathione S-transferase (GST), heme oxygenase 1 (HMO-1), or NADPH quinine oxidoreductase 1 (NQO-1), among others (Figure 5). Nuclear factor erythroid 2 related factor 2 (Nrf-2) is the transcription factor considered to be the main inductor of the Phase II genes, commonly called antioxidant-response element (ARE) genes. In addition, Nrf-2 forms a complex in the cytoplasm with Keap-1 (Kelch-like epichlorohydrin-associated protein 1), which is a negative regulator of the Nrf-2/ARE pathway [199]. Phase III enzymes are responsible for the export of the final metabolites out of cells.

The first evidence of the capacity of chlorophyll compounds as inducers of mammalian Phase II cytoprotective genes was reported by Fahey et al. [200]. They measured the ability of different chlorophyll structures to induce NQO-1. Among them, chlorophyllins and, above all, copper chlorin *e_4_* ethyl ester (Figure 3) were the most potent inducers related to chlorophyll, pheophytin, or pheophorbide. The authors demonstrated that copper chlorin *e_4_* (disodium) can react with free sulfhydryl groups, suggesting that they can bind to Keap-1 and ARE to trigger Phase II gene transcription. These results were subsequently supported by a completely different approach. An evident increase in the expression of Nrf-2 and a decrease in Keap-1 were observed in the buccal pouch tissues of experimental animals when their diet was supplemented with chlorophyllins [196], in parallel with the immunohistochemical staining of their proteins. The activation of Nrf-2 was concomitant with a decrease in the expression levels of *CYP1A1* and *CYP1B1* (cytochrome P450 monooxygenase), Phase I genes, and induction of the expression of Phase II genes (NQO-1 and GST). NQO1 functions as a part of the oxidative-stress-induced cellular defense when GST, through reactive oxygen species, converts to glutathione. Chlorophyllin enhanced the nuclear translocation of Nrf-2 in mouse splenic lymphocytes in both a dose- and time-dependent manner [201]. In rats with breast cancer, chlorophyllin increased the GSH levels in the liver [202].

Another line of research comes from the assumption that chlorophyll *a* and its derivatives have been proposed to exert antidiabetic functions [203]. At a molecular level, chlorophyll *a* metabolites are retinoic X receptors (RXRs), known to alter insulin and glucose signaling and, consequently, decrease hyperglycemia, hypertriglyceridemia, and hyperinsulinemia [204]. Evidence of chlorophyll’s influence in insulin metabolism was obtained using the animal experimental model *Caenorhabditis elegans* [205]. Insulin ligands, through a cascade of several kinases, can inactivate the transcription factor forkhead box (DAF-16/FOXO), thereby blocking the transcription of target genes such as the *SOD-3* gene [206], which codifies superoxide dismutase. Chlorophyll-treated nematodes modify the nuclear translocation of DAF-16 and increase the expression of *SOD-3*, increasing their lifespan by up to 25%. Such anti-aging activity could be promoted by chlorophyll’s enhancement of the tolerance to oxidative stress. Upon the same pro-oxidant (Juglone) treatment, the survival rate of nematodes with the chlorophyll diet increased more than 200% compared to the control group.

## 5. Conclusions

There is a primary chemical core understanding of the antioxidant behavior of carotenoids and chlorophyll pigments, but emerging pieces of evidence point to a rich diversity of actions and effects, which are intricate and distant from any antioxidant chemical nature. This is particularly true in the case of carotenoids, but chlorophylls are also being included in this concept. A fine line exists between experimental protocols and a lack of biological context of results, so the researcher should always consider the barrier between the in vitro and in vivo scenarios. Additionally, the limitations of studies, particularly those related to dosage strategies, and sources of the antioxidant (pure standard vs. food extract) should be noted. This review provides literature-based knowledge with the aim of advancing the concept of antioxidants in the near future to applications in the real world. Thus, it is necessary to set the average spread of reliable in vivo oxidative biomarkers with which to monitor the onset of degenerative diseases, so that the influence of dietary antioxidants could be established more precisely. A real effort from academics, research centers, and policymakers is taking shape in this line. However, we should not lose sight of the inverse association between pigment-containing fruits and vegetables with risk for various chronic diseases. It is also crucial to consider the stage of a disease at which antioxidant pigments may impact its development and progression. In this regard, it seems that carotenoids and chlorophylls have gone from being considered bioactive to becoming biomarkers of the onset of diseases related to oxidative stress. The key to continued support of the pivotal role of pigments in providing cooperative action within the antioxidant defense system is to look for metabolites arising early from imbalances of oxidative stress homeostasis. Currently available metabolomic platforms are starting to build the basis for such knowledge, which is expected to set future lines of research.

## Figures and Tables

**Table 1:** Chemical methods applied to measure the antioxidant activity of chlorophylls and carotenoids.

Method	Advantages	Disadvantages	Mechanism
ORAC	Related to in vivo conditions because it uses a biologically relevant free radical source (peroxyl radical) [10]. To avoid underestimation of antioxidant capacity and to account for potential effects of secondary antioxidant products, the ORAC assay monitors the reaction for extended time intervals (≥30 min).	Limited to the measurement of antioxidant capacity against peroxyl radicals and hydroxyl radicals, not applicable against any other ROS [10,32]. Proteins in plasma or serum samples must be removed as they can mask the response [12].	HAT1: Decrease of luminescence of target compound by peroxyl radicals.
TRAP	Involves the initiation of lipid peroxidation by generating water-soluble peroxyl radicals and is sensitive to any chain-breaking antioxidants [10]. Particularly suitable for plasma samples [33].	Relatively complex and time-consuming, requiring a high degree of expertise and experience [10]. The long time required per sample (up to 2 h) can lead to instability of the electrode [34]. Not appropriate for direct measurement of liposoluble antioxidants’ TAC [33].	HAT: Decay of fluorescent target once peroxided.
FRAP	Simple, rapid, inexpensive, and robust and does not require specialized equipment [35].	False high values can be obtained as an electron-donating substance can contribute to the FRAP value even without antioxidant properties with redox potential less than that of the redox pair Fe (III)/Fe (II) [36].	SET: Formation of a reduced ferric compound
CUPRAC	Low cost, reagent stability and accessibility, and response to both hydrophilic and lipophilic antioxidants [37].	The selection of appropriate reaction time due to the complex mixture of antioxidants [35].	SET: Formation of a reduced cupric compound
DPPH	Fast and technically simple. Good repeatability and frequently used [38]. Existence of a linear correlation between the DPPH concentration and absorbance means the DPPH method can be applied to chromophoric compounds such carotenoids [39]. Can only be accomplished in organic solvents, typical for chlorophylls and carotenoids.	The maximum absorbance is very close to that of the carotenoids. Not appropriate for uncommon highly polar chlorophylls and carotenoids [40]. Sensitive to acidic pH. High stabilization periods [38].	Mixed-mode: Ability to scavenge DPPH radical
ABTS	Useful at different pH conditions. *Short stabilization times. *Applicable to geometric carotenoid isomers [41]. The activity of the decomposition products of the β-carotene isomers is double that of α-tocopherol [26]; β-carotene does not lose its antioxidant activity by degradation to long-chain decomposition products. Soluble in water and organic solvents. ABTS working solutions can be kept in the dark for 12 h and then used within 4 h [42,43].	The reaction depends largely on the oxidizing agent used [4].	Mixed-mode: Formation of an oxidized radical cation.

Abbreviations: ORAC: oxygen-radical-absorbance capacity; ROS: reactive oxygen species; TRAP: total radical-trapping antioxidant parameter; TAC: total antioxidant capacity; FRAP: ferric-reducing/antioxidant power; CUPRAC: cupric-reducing antioxidant capacity; DPPH: 2,2-diphenyl-1-picryl-hydrazyl-hydrate; ABTS: 2,2′-azino-bis(3-ethylbenzothiazoline-6-sulfonic acid); HAT: hydrogen-atom-transfer-based assays; SET: single-electron-transfer-based assays.

**Table 2:** Biomarkers approved by the European Food Safety Authority (EFSA).

Biomarker	Direct In Vivo Measurement	Alternatives	Not Allowed
Protein	A method that allows separation and identification of oxidative changes in amino acids (e.g., protein tyrosine nitration products by LC-MS)	Protein carbonyls assessed by ELISA in blood or target tissue Conventional assays (e.g., colorimetric) in plasma samples	
Lipid	Changes in F2-isoprostanes in 24 h samples assessed using GC-MS or LC-MS Oxidized LDL particles in blood assessed using immunological methods Lipid hydroperoxides in blood or tissue assessed by CL-LC	Appropriate determination of MDA concentration in blood or tissues (e.g., LC)	In vivo TBARS In vivo MDA In vivo HDL-associated paroxonases In vivo conjugated dienes In vivo breath hydrocarbons In vivo LDL autoantibodies Ex vivo LDL resistance to oxidation
DNA	Modified comet assay (e.g., endonuclease III plus FPG)	Appropriate determination of urinary 8-OHdG (e.g., LC)	Traditional comet assay Ex vivo pro-oxidant comet assay

**Table 3:** Relevant studies considering the antioxidant capacity of carotenoids in liposomes.

Experimental Approach ^1^	Composition of the Membrane Model; Induction of Oxidative Stress; Biomarker(s)	Carotenoid(s)	Observed Effect	Reference
Lipid peroxidation in aqueous dispersion of lipids	Dimyristoil phosphatidylcholine liposomes or soybean phosphatidylcholine liposomes enriched with β-carotene; AAPH and AMVN peroxyl-radical inducers; phospholipid hydroperoxides, carotenoid bleaching	β-carotene	Potential of the antioxidant capacity of β-carotene is limited by the carotenoid autoxidation process that continues the chain oxidation of membrane lipids	[103]
Lipid peroxidation of liposomal bilayers	Dilinoleoylphosphatidylcholine liposomes enriched with β-carotene; AAPH radical inducer; conjugated dienes, carotenoid bleaching	β-carotene	β-carotene is an effective chain-breaking antioxidant when it is incorporated into the membranes	[104]
Lipid peroxidation in artificial membranes	Egg-yolk phosphatidylcholine liposomes enriched with a single carotenoid; AAPH and AMVN peroxyl-radical inducers; phospholipid hydroperoxides, carotenoid bleaching	Astaxanthin, β-carotene, canthaxanthin, lycopene, zeaxanthin; Antioxidant activity was compared with α-tocopherol	Different reactivity toward free radicals was correlated with the structural arrangement of the carotenoid, its position, and orientation in the bilayer	[105]
Lipid peroxidation in artificial membranes	Egg-yolk phosphatidylcholine liposomes enriched with a single carotenoid; UV radiation and AAPH peroxyl-radical inducer; TBARS, carotenoid bleaching	Lutein, zeaxanthin	Both xanthophylls are effective membrane antioxidants towards different radical induction processes; extension of lipid peroxidation was reduced by 35%; both xanthophylls promoted different changes in the organization of the lipids in the bilayer	[106]
Photoperoxidation of multilayer liposomes	Egg-yolk phosphatidylcholine liposomes enriched with individual carotenoid; hydrogen peroxide, *tert*-butyl hydroperoxide, ascorbic acid, and Fe^+2^-EDTA radical inducers; TBARS, iron concentration in liposomes	Astaxanthin, lycopene, peridinin	Structure of the carotenoids induced changes in the permeability of the membranes to radical inducers; a reduction of 25% of TBARS was observed after the addition of carotenoids to liposomes	[107]
Photoperoxidation of multilayer liposomes	Dimyristoil phosphatidylcholine/palmitoyl-oleoyl phosphatidylcholine/cholesterol liposomes enriched with individual or combined antioxidants; aerobic photo-peroxidation; lipid hydroperoxides, carotenoid bleaching, oxygen photo-uptake	Zeaxanthin	Zeaxanthin is an efficient membrane antioxidant toward singlet oxygen with a synergistic effect with α-tocopherol; Zeaxanthin is 30 times more effective than α-tocopherol in inhibiting photosensitized lipid peroxidation	[108]
Lipid peroxidation in unilamellar liposomes	Soybean phosphatidylcholine liposomes enriched with combined antioxidants; AAPH peroxyl-radical inducer; conjugated dienes	β-carotene, lycopene	Synergistic effects of carotenoids and tocopherols toward the lipid peroxidation process	[109]
Lipid peroxidation in unilamellar liposomes	Soybean phosphatidylcholine liposomes enriched with combined antioxidants; AAPH peroxyl-radical inducer; conjugated dienes	Astaxanthin	Synergistic effects of astaxanthin with both hydrophilic and lipophilic antioxidants were not significant	[110]
Lipid peroxidation in artificial membranes	Multilamellar liposomes enriched with PUFA/carotenoid; autoxidation; hydroperoxides, membrane interactions	Astaxanthin, β-carotene, lutein, lycopene, zeaxanthin	Apolar carotenoids behaved as membrane pro-oxidants, increasing the peroxide formation by 90–120%, whereas astaxanthin behaved as an antioxidant, decreasing the peroxide formation by 30%	[111]

^1^ Methods and experimental approach were developed by the authors, so the reader is advised to comprehensively review the studies included in this table. Abbreviations: AAPH: 2,2′-Azobis(2-methylpropionamidine) dihydrochloride; AMVN: 2,2′-azobis (2,4-dimethylvaleronitrile; TBARS: thiobarbituric-acid-reactive substances; PUFA: polyunsaturated fatty acids.

**Table 4:** Relevant studies dealing with the ex vivo antioxidant capacity of carotenoids in LDLs.

Experimental Approach	Participants; Supplementation Protocol; Biomarkers of Oxidative Stress; Relevant Methodologies	Carotenoids	Observed Effect	Reference
Supplementation/ex vivo LDL oxidation	Group of male nonsmokers and smokers; supplementation with β-carotene (2 × 20 mg daily for two weeks, and then 20 mg daily for 12 weeks); lipid peroxidation of LDL isolates [112]	β-carotene	No protective effect of LDL susceptibility to oxidation despite the observed increase in plasma β-carotene levels	[113]
Supplementation/ex vivo biomarkers of oxidative stress	Group of 11 smokers and 11 nonsmokers; supplementation with fruits and vegetables providing 30 mg carotenoids/day for 2 weeks; lipid peroxidation of LDL isolate supplementation, oxidative stress biomarkers of plasma [112]	α-carotene, β-carotene, lutein, lycopene, α-cryptoxanthin, β-cryptoxanthin	Inhibition of LDL susceptibility to oxidation for the smokers and nonsmokers. LDL resistance to oxidation increased 14% in smokers and 28% in the nonsmokers group after supplementation	[114]
Supplementation/ex vivo biomarkers of oxidative stress	Group of 32 healthy volunteers; double-blind randomized, placebo-controlled trial, supplementation with a mixture of carotenoids providing 7.6 mg carotenoids/day for 3 weeks; lipid peroxidation of LDL isolates, DNA damage, ORAC [8,112]	Lycopene, palm oil carotenes, marigold extract carotenoids, paprika carotenoids, bixin	The carotenoid supplementation reduced the LDL oxidizability (by 20.4% in the supplemented group) and DNA damage assessed by urine biomarkers; the effect was not observed with the ORAC assay	[115]
Supplementation/ex vivo biomarkers of oxidative stress	Group of 105 healthy volunteers; randomized, double-blind, placebo-controlled; commercial spread providing with a mixture of carotenoids at different doses for 11 weeks; lipid peroxidation of LDL isolates, plasma FRAP, MDA, serum arylesterase activity, plasma F_2__α_-isoprostanes; [28,112]	Lycopene, lutein, α-carotene, β-carotene	Moderate amounts of carotenoids resulted in a significantly increased resistance of LDL to oxidation and lower plasma peroxidation biomarkers (17% increase of LDL resistance to oxidation, 18% increase of lag-phase, and 15% reduction in the F2-isoprostane level)	[116]
Supplementation/ex vivo LDL oxidation	Group of 12 healthy female volunteers; supplementation with tomato products providing 8 mg lycopene/day for 21 days; lipid peroxidation of LDL isolates, urinary 8-iso-PGF_2__α_ [112,117]	Lycopene	Decrease in LDL oxidizability (22%) and significant lower excretion of 8-iso-PGF_2__α_ (53%) regarding the values reached in the control group	[118]
In vitro loading of LDL/biomarkers of oxidative stress	Group of 10 volunteers donated plasma samples for LDL isolation; in vitro loading was performed with lycopene or lutein via emulgent and incubation; lipid peroxidation and oxidation of ApoB of LDL isolates [108]	Lycopene, lutein	Carotenoids were not effective antioxidants of the LDL	[119]
	Group of 35 patients with T2DM; double-blind, placebo-controlled; supplementation with lycopene, 10 mg/day for 8 weeks; total antioxidant capacity assessed via ABTS, MDA, humoral immunity biomarkers	Lycopene	Increased ratio of total antioxidant capacity to MDA values and attenuated pro-atherogenic immune response	[120]
Supplementation/ex vivo LDL oxidation	Group of 77 healthy male and female volunteers; double-blind randomized, placebo-controlled trial, lycopene supplement at different doses for 8 weeks; lipid peroxidation of LDL isolates, MDA and HNE, urinary 8-iso-PGF_2__α_, DNA damage markers	Lycopene	Significant decrease in DNA damage (8.9%) and urinary 8-iso-PGF_2__α_ levels (23%) in the supplemented group; no significant effect was observed in biomarkers of lipid peroxidation	[121]
Supplementation/ex vivo biomarkers of oxidative stress	Group of 126 healthy men; randomized placebo-controlled trial, lycopene supplementation at different doses for 8 weeks; SOD activity in plasma, DNA damage, biomarkers of endothelial function	Lycopene, β-carotene	Increase in SOD activity (2.37 units/mL) and prevention of DNA damage (for the 15 mg/day suppl. group); beneficial effects in subjects with relatively impaired endothelial cell function	[122]

Abbreviations: SOD: superoxide reductase; suppl.: supplemented; T2DM: type 2 diabetes mellitus.

**Table 5:** Relevant studies dealing with the antioxidant capacity of carotenoids in cell systems.

Experimental Approach	Cell Model; Induction of Oxidative Stress; Biomarker(s)	Carotenoids	Observed Effect	Reference
Cellular membrane oxidation	Human HepG2 cells; *tert*-butyl hydroperoxide; lipid peroxidation and cellular leakage of lactate dehydrogenase	Micellar β-carotene (1.1 μmol/L) or lutein (10.9 μmol/L)	Protection of cellular membrane toward oxidant-induced changes	[133]
Lipid peroxidation	Normal and tumor thymocytes; AAPH and xanthine/xanthine oxidase, at low or high pO_2_; MDA and conjugated dienes	β-carotene in THF (6.3 mg/mL) to yield 10 μM–20 μM carotenoid concentration	Oxygen tension was a significant factor of β-carotene antioxidant efficiency. Lipid peroxidation rate increased 2.2-fold and 1.8-fold at 760 mm Hg pO_2_	[134]
DNA damage	HT29; xanthine/xanthine oxidase; oxidation of DNA and membrane integrity	Lycopene, β-carotene in THF to yield 1–10 μM concentration	Protection of oxidatively-induced DNA damage and membrane integrity Mean relative tail moment was reduced a 50% at 2.5 μM carotenoid concentration	[135]
Lipid peroxidation and DNA damage	CV1-P monkey cells; ferric nitrolotriacetate plus ascorbate; lipid peroxidation (TBARS) and 8-oxodGuo	Lycopene in THF to yield 3 mM concentration	Protection of mammalian cells against membrane and DNA damage. A 77% reduction in 8-oxodGuo level in lycopene-treated cells	[136]
Lipid peroxidation and DNA damage	Hs68 human foreskin fibroblasts; AAPH and AMVN and ferric nitrilotriacetate; lipid peroxidation, 8-OH-dG, comet assay	Lycopene, β-carotene in THF to yield 10 or 20 μM concentration	Both carotenoids performed antioxidant and pro-oxidant actions depending on the source of oxidative damage	[137]
UVA-photoprotection	Human skin fibroblasts; UVA radiation; metalloprotease 1 mRNA	Lycopene, β-carotene (nanoparticle formulation)	Reduction of the biomarker was only observed in the presence of vitamin E. Only a small induction of HO-1 was observed (1-2-fold) for lycopene or β-carotene treatments	[138]
Cellular membrane oxidation	Rat pheochromocytoma PC-12; deprivation of essential nutrients; peroxidation of membrane lipids and SOD activity	Crocin (0.1–10 μM)	Crocin was able to function as a chain-breaking antioxidant, restoring SOD activity (54% of the normal values) and maintained 60% of the neuron morphology	[139]
Oxidative stress of photoreceptors	Culture of rat retinal neurons; induced oxidative stress by paraquat and H_2_O_2_; apoptosis, mitochondrial membrane potential, cytochrome *c* translocation, and opsin expression	Lutein, zeaxanthin and β-carotene in 0.05% Tween solution	Carotenoids reduced the oxidative-stress-induced apoptosis as well as the other evaluated biomarkers. The 2.5-fold increase in photoreceptor cell death was suppressed in carotenoid-treated cells	[140]
Hydrogen peroxide damage	Rat erythrocytes (ex vivo); hydrogen-induced hemolysis; lipid peroxidation of membrane	Peel extracts of unripe and ripe mango fruits containing carotenoids. 5–25 μg of gallic acid equivalent in PBS	Protection against membrane protein degradation and morphological changes. 50% hemolysis inhibition was obtained at 11.5–20.9 μg GAE	[141]
UVA-photoprotection	Human dermal fibroblasts; UVA radiation; reactive oxygen species, apoptosis cascade enzymes, heme oxygenase expression	Astaxanthin, canthaxanthin, and β-carotene in THF to yield 0.5–10 μM concentration	Astaxanthin exerted a higher protective effect towards photo-oxidative damage. Measured ROS decreased by 30% and 50% in cells treated with astaxanthin at 5 μM	[142]
Functional integrity and mitochondrial redox state	Transfected HeLa human cervical cancer cells; hydrogen peroxide; redox-sensitive fluorescent protein imaging recording, mitochondrial membrane potential, superoxide levels	Astaxanthin in DMSO to yield 800 nM concentration	Reduction of basal oxidative stress, maintenance of mitochondrial membrane potential, improvement of the mitochondrial redox state	[143]
Mitochondrial function	Human HepG2 cells; carotenoid induction of ROS; ROS observed by fluorescence microscopy	Zeaxanthin, lutein and their 3-dehydro- derivatives	Mitochondrial carotenoid-oxygenase degraded carotenoids to protect the organelle functionality	[131]
Retinal degeneration	Mouse retinal ganglion cells RGC 5; tunicamycin, hydrogen peroxide; cell death, apoptosis cascade enzymes, nuclear layer thickness	Crocetin (0.1% in DMSO/PBS) to yield 0.1–3 μM concentration	Protective effects against retinal damage. Crocetin increased the protective effect against cell damage 5-fold	[144]
Photoprotective effect against UVB light	CCD-1064Sk human dermal fibroblasts; UVB irradiation; comet assay, UVB-induced cellular apoptosis	Capsanthin, capsorubin in THF/FBS to yield 1 μM concentration	The tested carotenoids decreased markers for UVB-induced apoptosis and interfered with cellular responses activated by UVB-mediated damage. DNA damage was decreased by 50% in capsanthin/capsorubin-treated cells after UVB irradiation	[145]

Abbreviations: MDA: malondialdehyde; THF: tetrahydrofuran; PBS: phosphate-buffered saline; DMSO: dimethyl sulfoxide; FBS: fetal bovine serum.

**Table 6:** Review of in vitro antioxidant activity measured for different free chlorophylls.

Method	Pigment	Concentration	Activity	Reference
β-carotene bleaching (% inhibition of oxidation)	Cu–chlorophyllin	681 μM	80%	[149]
	Pheophorbide *b*		80%	
	Pheophytin *b*		75%	
	Pheophorbide *a*		75%	
	Chlorophyll *a*		40%	
	Pheophytin *a*		70%	
β-carotene bleaching	Chlorophyll	0.05 μg/μL	49.63%	[165]
	Pheophytin		13.44%	
	Zn–pheophytin		66.43%	
β-carotene bleaching	Zn–chlorophyllin	5 mg/mL	82.00%	[170]
	Cu–chlorophyllin		74.40%	
	Iron–chlorophyllin		90.20%	
DPPH (% radical scavenging)	Cu–chlorophyllin	1 mM	39%	[149]
	Pheophorbide *b*	1 mM	<12%	
	Pheophytin *b*	1 mM	<12%	
	Pheophorbide *a*	1 mM	<12%	
	Chlorophyll *a*	1 mM	<12%	
	Pheophytin *a*		<12%	
DPPH	Pheophytin *a*	200 μM	55%	[171]
	Pheophytin *b*	200 μM	50%	
	Chlorophyll *a*	200 μM	40%	
	Chlorophyll *b*	200 μM	44%	
DPPH	Chlorophyll	0.05 μg/μL	13.89%	[165]
	Pheophytin		13.44%	
	Zn–pheophytin		66.43%	
DPPH	Zn–chlorophyllin	5 mg/mL	37.90%	[170]
	Cu–chlorophyllin		93.50%	
	Fe–chlorophyllin		26.50%	
DPPH (TEAC)	Pyropheophytin *a*	100 mM	0.02	[148]
	Pheophytin *a*		0.04	
	Pheophytin *b*		0.05	
	Chlorophyll *b*		0.06	
	Zn–Pheophytin *b*		0.13	
	Chlorophyll *a*		0.19	
	Pheophorbide *a*		0.21	
	Chlorin *e_4_*		0.26	
	Zn-Pyropheophytin *a*		0.44	
	Zn-Pheophytin *a*		0.51	
	Chlorin *e_6_*		0.6	
	Cu-Chlorin		0.81	
	Cu–Pheophorbide *a*		0.98	
	Cu–Pheophytin *a*		0.99	
	Crude SCC		1.04	
	Cu–Chlorin *e_6_*		2.88	
DPPH (I_50_)	Pheophorbide *a*		120 μM	[149]
	Pheophorbide *b*		75 μM	
	Chlorophyllin		360 μM	
	Chlorophyllide *a*		>800 μM	
	Chlorophyllide *b*		>800 μM	
DPPH (EC_50_)	Chlorin *e_6_*		23 μg/mL	[162]
ABTS (EC_50_)	Chlorin *e_6_*		52 μg/mL	[172]
ORAC (TEAC)	Chlorin *e_6_*	12.5 μg/mL	27 μM	[172]
ABTS (TEAC)	Pheophytin *a*	100 mM	0.02	[148]
	Pheophytin *b*		0.08	
	Pyropheophytin *a*		0.16	
	Chlorophyll *b*		0.23	
	Zn–pheophytin *b*		0.29	
	Zn–pheophytin *a*		0.43	
	Pheophorbide *a*		0.45	
	Chlorin *e_4_*		0.53	
	Cu–pheophytin *a*		0.58	
	Chlorin *e_6_*		0.64	
	Zn–pyropheophytin *a*		0.67	
	Chlorophyll *a*		0.73	
	Crude SCC		1.25	
	Cu–chlorin *e_4_*		1.35	
	Cu–chlorin *e_6_*		2.25	
	Cu–pheophorbide		2.4	
TBARS Kidney	Chlorophyll *b*	0.2 mg/kg b.w.	61.16 nmol/g	[173]
		0.5 mg/kg b.w.	62.06 nmol/g	
TBARS	Chlorophyll *b*	0.2 mg/kg b.w.	41.29 nmol/g	[173]
Liver		0.5 mg/kg b.w.	45.90 nmol/g	
Comet assay	Pheophytin *a*	50 μM	3500	[171]
	Pheophytin *b*	50 μM	3500	
	Chlorophyll *a*	50 μM	4000	
	Chlorophyll *b*	50 μM	4000	
Fe chelation	Pheophytin *a*	200 μM	65%	[171]
	Pheophytin *b*	200 μM	65%	
	Chlorophyll *a*	200 μM	55%	
	Chlorophyll *b*	200 μM	55%	
Lipid peroxidation	Pheophytin *a*	100 μM	75%	[171]
	Pheophytin *b*	100 μM	65%	
	Chlorophyll *a*	100 μM	95%	
	Chlorophyll *b*	100 μM	75%	
ROO· scavenging capacity (α-tocopherol relative)	Chlorophyll *a*		308	[171]
	Chlorophyll *b*		386	
Comet assay (Tail moment)	Cu–chlorophyllin	20 μM	138	[172]
	Chlorophyllide *a*	20 μM	136	
	Chlorophyllide *b*	20 μM	126	
	Pheophorbide *a*	20 μM	100	
	Pheophorbide *b*	20 μM	91	
8-OHdG (ng/μg DNA)	Cu–chlorophyllin	20 μM	0.53	[172]
	Chlorophyllide *a*	20 μM	0.68	
	Chlorophyllide *b*	20 μM	0.79	
	Pheophorbide *a*	20 μM	0.55	
	Pheophorbide *b*	20 μM	0.62	

Abbreviations: SCC: sodium copper chlorophyllins; b.w.: body weight; TEAC: Trolox-equivalent antioxidant capacity; I_50_: inhibitor concentration that causes 50% of inhibition; EC_50_: compound concentration that gives half-maximal response; TBARS: thiobarbituric acid-reactive substances assay; 8-OHdG: 8-hydroxy-2-deoxyguanosine.
